# The Associations of COVID-19 Lockdown Restrictions With Longer-Term Activity Levels of Working Adults With Type 2 Diabetes: Cohort Study

**DOI:** 10.2196/36181

**Published:** 2022-05-18

**Authors:** Christian John Brakenridge, Agus Salim, Genevieve Nissa Healy, Ruth Grigg, Alison Carver, Kym Rickards, Neville Owen, David Wayne Dunstan

**Affiliations:** 1 Baker Heart and Diabetes Institute Melbourne Australia; 2 Mary Mackillop Institute for Health Research Australian Catholic University Melbourne Australia; 3 School of Population and Global Health The University of Melbourne Melbourne Australia; 4 School of Human Movement and Nutrition Sciences The University of Queensland Queensland Australia; 5 Centre for Urban Transitions Swinburne University of Technology Melbourne Australia; 6 School of Exercise and Nutrition Sciences Institute for Physical Activity and Nutrition Deakin University Melbourne Australia

**Keywords:** COVID-19, Fitbit, activity, sedentary behavior, type 2 diabetes, digital health, pandemic, physical activity, wearable, health technology

## Abstract

**Background:**

Lockdown restrictions reduce COVID-19 community transmission; however, they may pose challenges for noncommunicable disease management. A 112-day hard lockdown in Victoria, Australia (commencing March 23, 2020) coincided with an intervention trial of reducing and breaking up sitting time in desk workers with type 2 diabetes who were using a provided consumer-grade activity tracker (Fitbit).

**Objective:**

This study aims to compare continuously recorded activity levels preceding and during COVID-19 lockdown restrictions among working adults with type 2 diabetes participating in a sitting less and moving more intervention.

**Methods:**

A total of 11 participants (n=8 male; mean age 52.8, SD 5 years) in Melbourne, Australia had Fitbit activity tracked before (mean 122.7, SD 47.9 days) and during (mean 99.7, SD 62.5 days) citywide COVID-19 lockdown restrictions. Regression models compared device (Fitbit Inspire HR)–derived activity (steps; metabolic equivalent tasks [METs]; mean time in sedentary, lightly, fairly, and very active minutes; and usual bout durations) during restrictions to prerestrictions. Changes in activity were statistically significant when estimates (Δ%) did not intercept zero.

**Results:**

Overall, there was a decrease in mean steps (–1584 steps/day; Δ% –9%, 95% CI –11% to –7%); METs (–83 METs/day; Δ% –5%, 95% CI –6% to –5%); and lightly active (Δ% –4%, 95% CI –8% to –1%), fairly active (Δ% –8%, 95% CI –21% to –15%), and very active (Δ% –8%, 95% CI –11% to –5%) intensity minutes per day, and increases in mean sedentary minutes per day (51 mins/day; Δ% 3%, 95% CI 1%-6%). Only very active (+5.1 mins) and sedentary (+4.3 mins) bout durations changed significantly.

**Conclusions:**

In a convenience sample of adults with type 2 diabetes, COVID-19 lockdown restrictions were associated with decreases in overall activity levels and increases in very active and sedentary bout durations. A Fitbit monitor provided meaningful continuous long-term data in this context.

**Trial Registration:**

Australian New Zealand Clinical Trials Registry ACTRN12618001159246; https://anzctr.org.au/Trial/Registration/TrialReview.aspx?ACTRN=12618001159246

## Introduction

The COVID-19 pandemic continues to have a lasting impact on the health care system [1,2]; as of December 2021, there have been 270 million confirmed cases since the pandemic began [3]. Type 2 diabetes mellitus is prevalent in patients admitted to hospitals with COVID-19 [4,5], with rates as high as 33.8% [6]. Poorer glycemic control can also be a predictor of COVID-19 mortality [7,8]. Regular physical activity is recognized as a cornerstone of diabetes management and glycemic control [9]. However, there is now evidence that some of the public health measures used to contain the spread of the virus, including restriction of movement via community-level lockdowns, may have impacted physical activity levels [10].

Specifically, there is evidence of pandemic-associated decreases in overall physical activity [11,12] and decreases in activity of different intensities (ie, light, moderate, and vigorous) [13,14], along with an increase in sedentary time [15]. For example, a study using hip-worn accelerometers found that sedentary behavior time and prolonged sedentary bouts increased and total daily steps decreased during lockdowns [16]. Collectively, these findings suggest that COVID-19 lockdown restrictions are likely to adversely impact a set of lifestyle behaviors important to the management of type 2 diabetes; however, the relevant evidence has some limitations. Most of these studies have used self-report measures of activity [17-19] with many involving cross-sectional designs [15,20] or using retrospective data collection [12,21]—design types that are prone to recall and reporting biases. In those studies where devices were used (eg, body-worn accelerometers), short time frames (7-8 days) were observed [16,22], which limits the opportunity to understand long-term trends. Aside from a few studies [23-26], most investigations have featured short or singular time frames of observation and, importantly, have not measured activity behaviors both immediately before and at the point of a COVID-19 outbreak. Furthermore, despite being recognized as a population at greater risk of the health impacts of COVID-19, no studies have assessed physical activity with continuously worn devices prior to and during COVID-19 in people with type 2 diabetes.

Prolonged and restrictive lockdown conditions imposed on residents living in Melbourne, Australia coincided with the conduct of a clinical trial in working adults with type 2 diabetes (ANZCTRN12618001159246), targeting both reductions in sedentary time and increases in physical activity. With one of the intervention components requiring participants to use a wrist-worn activity tracker throughout, this presented an opportunity to ascertain the impact of lockdown on these trial participants.

This exploratory study uses data from a wrist-worn consumer device (Fitbit) to describe and compare activity levels of working adults with type 2 diabetes participating in a behavior change intervention trial prior to and during a prolonged citywide lockdown due to COVID-19.

## Methods

### Participants and Setting

Participants were from the intervention arm of the OPTIMISE Your Health trial [27]. This trial, which began in 2019, aims to both reduce sedentary behavior (sit less) and increase movement (move more) in desk workers with type 2 diabetes aged 35 to 65 years [28]. Eligibility is based on having been diagnosed with type 2 diabetes (confirmed with recent hemoglobin A_1c_ [HbA_1c_] test), not being on insulin therapy, working in a desk-based occupation (0.8-1 full-time equivalent), high sedentary time (>50% of waking hours), not meeting physical activity guidelines (ie, doing <150 min of moderate-vigorous physical activity/week and <2 strength sessions/week), and living within a 40 kilometer radius of the Baker Heart and Diabetes Institute (Melbourne, Australia). Participants were randomized following baseline assessments into control or intervention arms. Those in the intervention arm received a height-adjustable desk, a Fitbit Inspire HR wrist-worn fitness tracker, and behavior change health coaching support, as described in detail elsewhere [27]. In brief, the health coaching involved participants setting incremental goals to reduce sitting and increase physical activity, which was facilitated by behavioral strategies that encouraged self-management (eg, standing up after a work task or taking a light walk after finishing a meal). A convenience sample of 11 intervention-arm participants who wore the Fitbit both prior to and during the lockdown restriction periods were included in this study. Participants were recruited sequentially into the broader trial, hence Fitbit observation windows differed for each participant.

### Ethical Considerations

Protocols and ethics were approved by the Alfred Health Ethics Committee (#359/18), and all participants provided written informed consent.

### Data Collection

The baseline assessment included demographic questions, anthropometric measures (height, weight), and a fasting blood glucose examination. Two weeks following baseline assessment, participants were provided with their Fitbit Inspire HR device, which they were encouraged to wear as often as possible to promote and maintain physical activity behaviors. Participants were not required to wear the Fitbit while sleeping; therefore, sleep was not investigated. Participants consented to give access to their Fitbit activity (recorded on the study account) via Fitabase (Small Steps Labs LLC), a third party web-based data management platform. Each participant was set up with a unique Fitabase study account linked to their Fitbit device and associated smartphone app. All data synchronized from the wearable device to the Fitbit app was uploaded to Fitabase automatically where it could then be exported into date- and time-stamped minute intervals for the time period from August 10, 2019 (start of data collection for the first participant) until October 18, 2020 (the end of the first major lockdown period).

### COVID-19 Lockdown in Melbourne, Australia

The first COVID-19 lockdown restrictions in Melbourne, Australia commenced on March 23, 2020, and were eased intermittently, then reinstated, and not lifted until October 18, 2020. During this time, Australia was ranked as having one of the strictest pandemic mitigation strategies in the world, reaching a high of 80 in a 1 to 100 stringency scale in March 2020 [29]. Of all Australian cities, Melbourne had the strictest lockdown during this time due to high rates of transmission and the state government’s intention for complete elimination of community transmission of the virus. Varying restrictions were imposed in Melbourne throughout the period of observation; these are described in greater detail in Multimedia Appendix 1.

The lockdown restrictions led to the OPTIMISE Your Health trial being placed on temporary hold. This entailed the suspension of recruitment and clinical assessment visits. Following this, participants were sent a survey via email with a series of questionnaires enquiring about any changes incurred due to the pandemic. The questions pertained to changes in work hours; work location; sitting, standing, and activity behaviors at work; workload; care load; physical activity and exercise; sedentary behavior; motivation; work environment; and musculoskeletal health. Enrolled participants who were involved in the trial during the lockdown period were encouraged to complete their participation to the end of the original trial date (6 months).

### Fitbit-Derived Activity Metrics

The Fitbit Inspire HR is a wrist-worn, triaxial accelerometer. The device also uses photoplethysmography, which measures heart rate with infrared light through the skin. The device is powered by a lithium polymer battery with an average battery life of 5 days depending on use. A proprietary algorithm converts the raw acceleration signal from the tracker into step counts and activity intensity. Each minute interval is categorized as sedentary (<1.5 metabolic equivalent tasks [METs] according to Fitbit), lightly active (1.5-3 METs), fairly active (3-6 METs), or very active (>6 METs or ≥145 steps/min in at least 10 min bouts) according to Fitbit’s determination of METs [30]. Data were collected continuously by the wrist-worn tracker for 30 days, at which point the device must be synchronized via Bluetooth with a smartphone and the Fitbit app. For the OPTIMISE Your Health trial, the provision of the Fitbit allowed participants to monitor their activity behaviors on the device and the smartphone app. Participants were encouraged to self-select daily stepping goals and activity break reminders (up to 14 per day) that prompted them to achieve 250 steps in each hour of the day.

A recent validation study determined that the Fitbit Charge 2, an older model, demonstrated high correlation (intraclass correlation coefficient >0.89) with an established research-grade accelerometer (Actigraph GT1X) in free-living observations [31]. In that study, correlations between the Fitbit Flex and GT3X+ data were high for sedentary time (*r*=0.9) but weaker and overestimated for moderate-vigorous intensity physical activity (*r*=0.65-0.76) [32]. A systematic review in 2016 featuring analysis of 13 studies examining the accuracy of Fitbit in free-living conditions determined that the Fitbit had a tendency to overestimate steps (700-1800 steps/day) compared to research-grade devices [33]. There is currently no validation study published for the Fitbit Inspire HR used in this trial.

### Statistical Analyses

For each study participant, the following data was downloaded via Fitabase for the entire wear period: daily steps, METs, heart rates, and estimated daily sleeping time (if available). Steps, METs, and heart rate data are available in 1-minute resolutions, and the associated time stamps are available for all variables. Prior to analyses, all data where the time stamps matched at least one of these criteria were removed: corresponded to time intervals detected as sleeping time by Fitbit, was between midnight and 5 AM daily, and time stamped with a heart rate reported as 0. The first two criteria were used to remove segments that correspond to sleeping time, and the third was used to remove segments when the Fitbit was not worn. This defined the daily waking period. The remaining data were analyzed with models fitted for each participant, separately for METs per minute, the intensity minute categories (sedentary, lightly active, fairly active, very active intensity mins), and step counts. For METs-based analysis, data were analyzed at 1-minute resolution with the logarithm of each day determined as a METs per day–dependent variable. For the intensity-based analyses, the log average number of minutes spent in each intensity category was used as the dependent variable. For steps-based analysis, the logarithm of the daily number of steps was used as the dependent variable. These dependent variables were log transformed to improve the normality of residuals in the model and to ensure nonnegative predicted values following back transformation. The usual bout duration, also known as the weighted median statistic (w50 or x50), was calculated for all activity intensities according to a previously devised method [34]. This entailed all bouts being ordered according to bout duration (mins) and normalized as a proportion of total time spent in each activity intensity type. Participants accumulated half of all their activity time in bouts longer than their usual bout duration.

We used fixed-effect meta-analysis to combine the regression coefficient with lockdown effects into a pooled result for all participants. The METs per minute and intensity minutes models were fitted using generalized least squares regression with autoregressive error structure to handle within-individual autocorrelation. Step counts were fitted with negative binomial regression methods. For all models, the main independent variable was the lockdown time indicator. This indicator included two states: before lockdown (before March 23) or during lockdown (on or after March 23). Independent variables were also added to adjust for differences in Fitbit wear habits that may have occurred following lockdown restrictions: these were calculated as sin(2π**t**/24) and cos(2π**t**/24), where **t** was the time stamp in a 24-hour continuous time format, and the interaction between lockdown time indicator and sin and cosine terms was modeled. To determine the average absolute change following restrictions, steps and METs were transformed from hour and minute intervals to per day for ease of interpretation. For all analyses, the main parameter of interest was the antilog of the regression coefficient associated with the lockdown variable; this was interpreted as relative rates, with the prelockdown period considered the reference. Relative rates were then transformed into percentages (Δ%). A statistically significant difference between the lockdown period and the prelockdown period was determined when Δ% did not intercept zero.

A postpower calculation was performed using the R Package “PASSED” version 1.2.1 [35] for daily step count. For this analysis, it was assumed that steps per day followed a negative binomial distribution with the distribution statistic (theta) set at 5. Given a mean daily step count of 10,000 steps prelockdown restrictions and a minimum of 100 days prelockdown and 100 days following lockdown restrictions, there was at least 80% power to detect a 10% or more reduction in step count for 11 participants.

## Results

### Sample Characteristics and Period of Observation With Fitbit in the COVID-19 Pandemic

**Figure 1:**
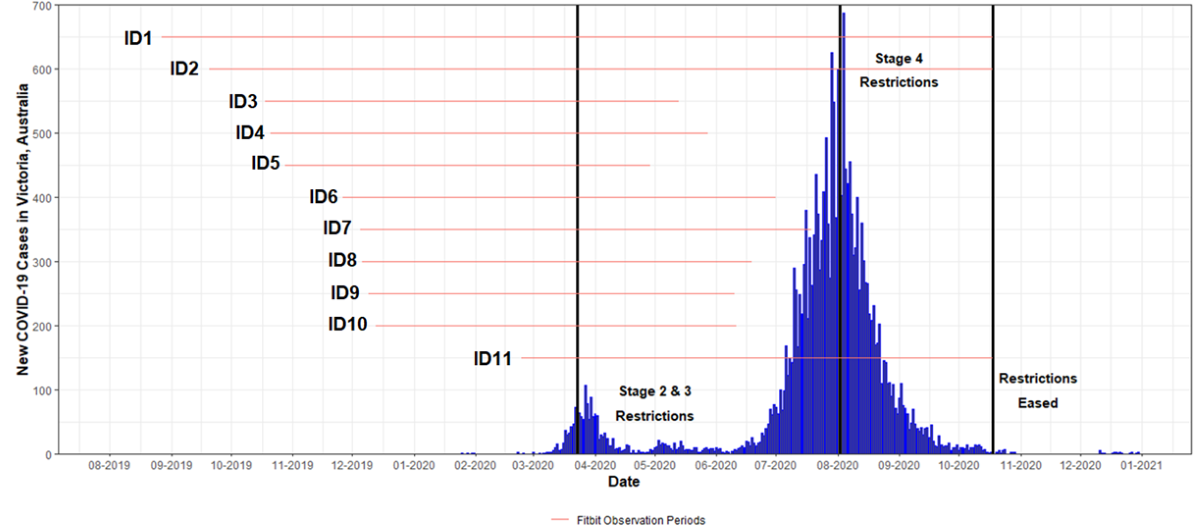
New COVID-19 cases in the months preceding and during the 2020 pandemic in Melbourne, Victoria, Australia. Fitbit observation period for each participant is depicted by red lines. Stage 2 restrictions (initiated March 23) entailed shutdown of all nonessential businesses and activities. Stage 3 restrictions (initiated March 30) enforced that people only leave their homes for four reasons: food and supplies, medical care, exercise, and work or education. Gatherings of no more than two people were allowed outside unless they were members of an immediate household or if it was for work or education purposes. Stage 4 restrictions (initiated August 2) included Stage 3 restrictions and the addition of a nightly curfew 7 PM to 5 AM, mandatory face coverings in public, the closing of schools and businesses, and a 5 km radius (around the home) for exercising and essential shopping. On October 18, 2020, the radius of restriction increased to 25 km, 10 people from 2 households allowed to gather in outdoor spaces, and businesses allowed to reopen with conditions. Novel case data and timeline extracted from Victorian Department of Health and Human Services data dashboard [36].

The mean age of the 11 participants was 52.8 (SD 5) years, and the majority were male (n=8, 73%). In line with the trial inclusion criteria, participants were overweight/obese (mean BMI 35.2, SD 5.1 kg/m^2^), with a mean HbA_1c_ of 7.6% (SD 0.8%) at the commencement of their trial participation. A timeline of stage 2, 3, and 4 COVID-19 lockdown restrictions that entailed stay-at-home orders are summarized with novel case data for the state of Victoria (capital city: Melbourne) in Figure 1. According to questionnaire findings (Multimedia Appendix 2), following the imposed restrictions, participants (n=9) reported a shift toward working from home more, a less desirable workplace environment, and reductions in physical activity and exercise participation. None of the participants reported having a COVID-19 infection or having to self-isolate as a close contact during the period of observation. Timelines of Fitbit data collection were reported for each participant. A total of 2447 wear days were recorded across the 11 participants with a median of 197 (range 167-418) days per participant. All participants had substantial periods of observation prior to (mean 122.7, SD 47.9 days) and during the lockdowns (mean 99.7, SD 62.5 days).

### Comparison of Activity Minutes Identified by the Fitbit During and Prior to Lockdown

In the overall pooled-analysis of the participants’ activity levels (Table 1), it was determined that both steps (absolute change –1584; Δ% –9%, 95% CI –11% to –7%) and METs per day (absolute change –83; Δ% –6%, 95% CI –6% to –6%) decreased under lockdown restrictions compared with prerestriction levels. Lightly active minutes (absolute change –11 mins; Δ% –4%, 95% CI –8% to –1%) and fairly active minutes (absolute change –3 mins; Δ% –18%, 95% CI –21% to –15%) decreased following the restrictions, and there was a gain to sedentary minutes (absolute change 51 mins; Δ% 3%, 95% CI 1%-6%). Minutes of very active intensity decreased (absolute change –5 mins; Δ% –8%, 95% CI –11% to –5%); however, the usual bout duration of the very active bouts increased (absolute change 5.1 mins; Δ% 25%, 95% CI 4%-49%). Usual sedentary bout duration also increased (absolute change 4.3 mins; Δ% 20%, 95% CI 16%-25%). There were minimal changes to lightly active and fairly active intensity usual bout durations following restrictions, with estimates not reaching statistical significance.

**Figure 2:**
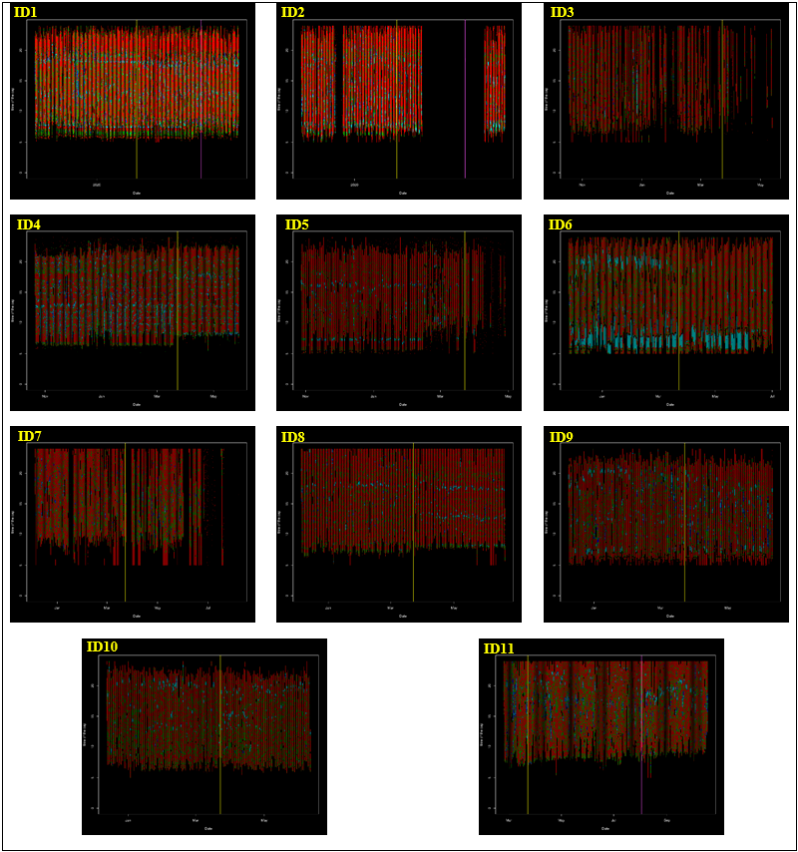
Minute-by-minute physical activity intensities during the period of Fitbit observation. Heat maps are numbered according to the participant. Red=sedentary minute, green=lightly active minute, blue=fairly active minute, cyan=very active minute, black=Fitbit not worn/sleep period. The yellow vertical line indicates the start of the lockdown period and stage 2 and 3 restrictions on March 23, 2020; the fuchsia vertical line indicates stage 4 restrictions that started on August 2, 2020.

In the individual participant analysis (Multimedia Appendix 3), there was evident heterogeneity in the participants’ responses to lockdown restrictions. Considering the 11 participants individually, 4 increased their mean daily step counts with 2 of these participants also increasing their METs. The increases made to activity levels by these participants were outweighed by the remaining sample that saw a decrease in activity for steps (mean increase 575 steps; mean decrease 2760 steps) and for METs (mean increase 43 METs; mean decrease 144 METs). Discrepancies between changes in step counts and energy expenditure occurred due to differing engagement in activity intensities following the restrictions. Of all participants, 3 increased lightly active intensity minutes, 4 increased fairly active intensity minutes, 5 increased very active intensity minutes, and 5 decreased sedentary minutes per day. The most consistent changes at the individual level were increases to usual sedentary bout durations with 10 participants (9 statistically significant) increasing their volume of time spent in sedentary bouts. Similarly, 7 participants increased usual very active intensity bout durations, although only 2 had statistically significant within-individual changes. The individual responses to the lockdown restrictions are depicted in the Figure 2 heat map visualizations for each participant.

**Table 1:** Activity conducted during lockdown restrictions compared to activity conducted prior to lockdown restrictions.

		Overall pooled estimates			
		Prior to lockdown restrictions, mean (SD)	During lockdown restrictions, mean (SD)	Difference	Δ%^a^ (95% CI)
**Total activity per day**					
	Steps (n/day)	10,623 (4439)	9039 (3351)	–1584	–9 (–11 to –7)
	METs^b^ (n/day)	1940 (264)	1857 (173)	–83	–5 (–6 to –5)
	Lightly active intensity (mins/day)	251 (6)	240 (6)	–11	–4 (–8 to –1)
	Fairly active intensity (mins/day)	16 (0)	13 (1)	–3	–18 (–21 to –15)
	Very active intensity (mins/day)	32 (1)	27 (2)	–5	–8 (–11 to –5)
	Sedentary (mins/day)	1064 (25)	1115 (36)	51	3 (1 to 6)
**Usual bout duration^c^**					
	Lightly active intensity (mins)	4.4 (0.5)	4.5 (0.9)	0.1	1 (–4 to 7)
	Fairly active intensity (mins)	2.7 (0.6)	2.5 (0.7)	–0.2	–7 (–19 to 6)
	Very active intensity (mins)	15.7 (20.2)	20.8 (25.7)	5.1	25 (4 to 49)
	Sedentary (mins)	20.2 (6)	24.5 (7.6)	4.3	20 (16 to 25)

^a^Δ%: change in activity following pandemic lockdown restrictions.

^b^MET: metabolic equivalent task.

^c^Usual bout duration describes the median weighted bout length; participants accumulate half of all their activity time in bouts longer than the estimate.

## Discussion

### Principal Findings

Participants with type 2 diabetes involved in an intervention targeting sitting less and moving more wore a consumer-grade activity-monitoring device (Fitbit Inspire HR) that identified overall decreases in active time and increases in sedentary time following COVID-19 lockdown restrictions. These changes were characterized by a decrease in time spent in lightly active, fairly active, and very active physical activity intensities, and an increase to time spent sedentary. Combined, these behavioral changes would be expected to have adverse implications for glycemic control and diabetes management.

Findings from this study corroborate current evidence on changes to physical activity associated with the COVID-19 pandemic [10] and confirm findings published online by Fitbit in early 2020 [37]. They also align with observations that those who are overweight or obese were also likely to have their physical activity levels adversely affected by the pandemic [23]. In Spain, a study of 72 patients with diabetes self-reported a significant decline in their weekly walking time during lockdown restrictions [38]. Examining data from a cohort similar to those in our study, a recent study [39] featuring people with type 2 diabetes living in Melbourne, Australia found that self-reported total physical activity did not change, but incidental walking decreased. For this study, less incidental activity could have contributed to the observed prolonging of sedentary bouts. This could be due to a number of reasons such as minimizing movement to reduce chance of transmission in the community [40], anxiety leaving the house [41], a reduction in physically active commuting [42], and widespread changes to permitted activities in the neighborhood environment [43]. It has previously been reported that fitness-oriented walking was surmised to have increased due to it being designated as one of the permissible reasons to leave home during restrictions [39]. More purposeful fitness-based walking may have explained the slight increase in very active intensity bout duration found in our Fitbit analysis. However, the modest increases to average time spent in very active intensity bouts were not sufficient to counter the overall decline in total activity in the pooled analyses.

### Comparison With Prior Work and Implications for Future Research

In the context of a continuing pandemic that may involve future restrictions, we have identified the need to proactively address sedentary behavior reduction and the promotion of increased physical activity (even light-intensity physical activity) in people with type 2 diabetes [44]. The overall decline in step counts observed in this study have potential implications for health outcomes. Based on previous observational research findings, a reduction of 500 steps per day in inactive people is associated with an approximate 2% to 9% increased risk of cardiovascular morbidity and all-cause mortality, and is associated with a 5% increase in all-cause mortality risk when measured by wrist-worn devices [45]. These extrapolations are important for people with type 2 diabetes who are already at heightened risk of cardiovascular disease and morbidity [46]. Conversely, maintaining physical activity levels is associated with a lower susceptibility to viral infections such as COVID-19 [47], improved vaccine efficacy [48], and reduced odds of hospitalization with severe COVID-19 outcomes [49]. Therefore, during public health crises like a pandemic, physical activity levels should be monitored to inform policy that strikes a balance between disease mitigation and the maintenance of physical activity participation in the community [50].

Here, we used a Fitbit device to evaluate the effects of COVID-19 restrictions, with the overall findings indicating a decline in activity levels largely corroborated by other recent evidence. It should, however, be acknowledged that some people within this analysis succeeded in either increasing their activity or decreasing sedentary time despite the lockdown restrictions. For example, the heat map visualizations in Figure 2 illustrate that both ID6 and ID11 increased their very active intensity bout lengths when they engaged in them, while ID10 spent less time in unbroken sedentary bouts that contributed to the preservation of their activity levels. For ID7, there was a significant increase in time spent in lightly active intensities and an increase in lightly active bout durations following the restrictions. While the findings overall indicate a negative impact of the lockdown restrictions, understanding how some participants maintained or improved their activity levels may inform intervention approaches and recommendations for subsequent lockdown restrictions and preventative measures.

Beyond the pandemic, there is potential for consumer-grade devices to be used for measurement in research studies [51], especially considering their ubiquity in society and constant technological advancement. Importantly, these devices can capture physical activity data over longer periods of time than those achieved by traditional research-grade activity monitors that typically measure 7 to 14 days of data. However, consumer-grade devices need to be validated against measures derived from traditional research-grade monitors, and comparisons made between short-term and longer-term periods of physical activity measurement. There is the potential for consumer-grade devices to be used in determining physical activity adherence and the effects of interventions (eg, following physical activity or dietary intervention) or to investigate longer periods of activity and the relevance to long-term factors of diabetes management such as glycated hemoglobin, adiposity, or diabetes complications. Consumer-grade continuous measurement devices have already been used to prompt behavior change and improve glycemic control [52], and there may be added benefit through combining their use with continuous glucose monitors.

### Strengths and Limitations

This is one of only a few studies [23,53] that has used a continuous objective measure of physical activity to determine activity levels prior to and during the COVID-19 pandemic lockdown restrictions and the first to use this methodology in a sample of patients with type 2 diabetes. Using a Fitbit wrist-worn activity tracker, over 2000 wear days were recorded measuring physical activity continuously via accelerometry and heart rate, collected at 1-minute intervals. A wrist-worn device permitted enhanced capturing of physical activity levels when compared to a smartphone app, especially when confined to the home setting. With regression modeling, we were able to investigate the prospective associations of lockdown restrictions on activity levels.

Although we used an advanced method of analysis with high-resolution data with hundreds of wear days per person, we were limited by a small sample size, thus the findings are exploratory. Further participant recruitment was not feasible with pandemic restrictions, and it was necessary to restrict the selection of participants to a period in which they were exposed to comparable lockdown measures. Therefore an a priori power analysis to estimate necessary sample size was not pragmatic. The findings may have limited generalizability to the broader population of adults with type 2 diabetes. For instance, our participants were involved in an intervention trial in which they received coaching and tools to increase activity and reduce sedentary behavior, which became suspended because of restrictions. As the control group was not provided a Fitbit, the influence of the intervention could not be differentiated. One possibility is that the intervention could have provided protection from even further declines in activity level. Nevertheless, the observation that most of these intervention participants did not manage to keep their current activity levels may illustrate the substantial impact of the lockdown restrictions. All participants had type 2 diabetes, and while having relatively good management, evidenced by their levels of glycemic control, they had low baseline levels of physical activity that may have predisposed them to have greater changes induced by the restrictions. Another consideration is that, compared to other cities and countries, Melbourne and Australia had stringent lockdown restrictions. This means that these findings may not apply to other jurisdictions with less severe restrictions. Finally, the Fitbit is uniquely able to characterize longer-term physical activity; however, the model (Inspire HR) that we used does not have a validation study supporting it. Future studies are now required to corroborate these findings with research-grade measures and to better understand the potential for Fitbit to characterize physical activity over long observation periods.

### Conclusions

For participants with type 2 diabetes enrolled in an intervention trial to reduce sitting time and increase daily physical activity, the COVID-19 pandemic and subsequent lockdown restrictions led to a decrease in steps; METs; and lightly active, fairly active, and very active physical activity intensities, and an increase in time spent in very active and sedentary bout durations overall; however, there was wide individual variation. Data from the wrist-worn Fitbit consumer device provided interpretable long-term activity data to be able to examine these activity patterns. Further corroboration using concurrent data from research-grade measures is required.

## Appendix

Multimedia Appendix 1 COVID-19 pandemic restrictions timeline in Melbourne, Australia, 2020.

Multimedia Appendix 2 Self-reported changes in work, physical activity, sedentary behaviour, motivation, and musculoskeletal discomfort following COVID-19 pandemic lockdown restrictions.

Multimedia Appendix 3 Linear regression activity comparisons prior to lockdown restrictions and during for each participant.

## Abbreviations

HbA_1c_: hemoglobin A_1c_
MET: metabolic equivalent task
