# Supplementary material for: The Associations of COVID-19 Lockdown Restrictions With Longer-Term Activity Levels of Working Adults With Type 2 Diabetes: Cohort Study
Source: JMIR Diabetes. 2022 May 18;7(2):e36181. doi: 10.2196/36181 (PMC9119394; doi:10.2196/36181)
Supplement: Multimedia Appendix 1 [file diabetes_v7i2e36181_app1.docx]

## Multimedia Appendix

### *Multimedia Appendix 1– COVID-19 pandemic restrictions timeline in Melbourne, Australia, 2020*

| Table 1 – COVID-19 pandemic restrictions timeline in Melbourne, Australia, 2020 | |
| --- | --- |
| Date Imposed | Restriction Details |
| 16 March 2020 | Victorian government announced a “State of Emergency” for four weeks, the first direction was made banning non-essential gatherings of over 500 people. |
| 18 March 2020 | Indoor public gatherings of more than 100 people banned and further restrictions added. |
| 23 March 2020 | Stage 2 -Initiation of Australia-wide Stage 2 restrictions with shutdown of all non-essential businesses and activities. |
| 30 March 2020 | Stage 3 - restrictions allowed people to only leave their homes for four reasons: food and supplies; medical care; exercise; and work or education. Gatherings of no more than two people unless they were members of immediate household and it was for work or education purposes. |
| 12 April 2020 | State of emergency in Victoria extended by an additional four weeks until 11 May 2020. |
| 11 May 2020 | Slight easing to maximum gatherings of up to ten people outdoors and five visiting a home and return of some outdoor recreational activities. |
| 21 June 2020 | Announcement that the state of emergency to be extended by another four weeks until 19 July 2020 |
| 30 June 2020 | Stage 3 - Specific postcodes returning to stage 3 - stay at home restrictions |
| 9 July 2020 | Stage 3 - lockdown re-introduced for entire metropolitan Melbourne and Melbourne shire |
| 22 July | Face masks introduced for Melbourne at all times outside of the home. |
| 2 August 2020 | Stage 4 - State of disaster declared with increased restrictions: nightly curfew 7pm-5am, mandatory face masks in public and closing of schools and businesses, limited 5km radius around home for exercising and essential shopping. Exercise outside maximum of 1 hour per day. |
| 13 September | Curfew reduced to 9pm-5am, exercise increased to 2 hours per day |
| 5 October | VCE students return to school in masks |
| 12 October | Curfew ceased , prep to primary school students return to school in metropolitan Melbourne, |
| 18 October | 25km restriction, 10 people from 2 households in outdoor spaces, no longer time restrictions for outdoor activities, business allowed to reopen ‘if you can work from home you must work from home’ |
| 26 October | all other school students to return to face to face teaching, cafes reopening with caps for face to face dining, weddings and funerals increase capacity. Return to work allowed, essential workers permits no longer needed |
| 8 November | Metropolitan/regional border removed. 2 visitors to the home, masks at all times outside the home |
| 22 November | Mask at all times outside the home, 10 visitors to the house, public gatherings of up to 50 |
| 30 November | 25% can work in the office, masks only on public transport and indoor venues |
| 14 December | Household party caps 30 people, contact sports recommence, increased entertainment caps |
| Dates and restriction details summarised from official Australian Parliament website and the Department of Health and Human Services updates archive (1) | |

References:

1. Department of Health and Human Services. Coronavirus (COVID-19) Updates Archive [Internet]. 2020 [cited 2021 Jul 12]. Available from: https://www.dhhs.vic.gov.au/coronavirus/updates/202011
