# Supplementary material for: The Associations of COVID-19 Lockdown Restrictions With Longer-Term Activity Levels of Working Adults With Type 2 Diabetes: Cohort Study
Source: JMIR Diabetes. 2022 May 18;7(2):e36181. doi: 10.2196/36181 (PMC9119394; doi:10.2196/36181)
Supplement: Multimedia Appendix 2 [file diabetes_v7i2e36181_app2.docx]

|  |  |
| --- | --- |
| Self-report work factors prior to COVID-19 | Sample n=9 |
| Work hours, *mean (sd)* | 40.0 (3.9) |
| Work location |  |
| Work from home, *n (%)* | 1 (11.1) |
| Mostly work from home, *n (%)* | 0 |
| About equal work from home and workplace, *n (%)* | 0 |
| Mostly work at workplace, *n (%)* | 2 (22.2) |
| At workplace, *n (%)* | 6 (66.7) |
| Self-reported behaviours in workday |  |
| Sitting, *% (sd)* | 51.1 (24.1) |
| Standing, *% (sd)* | 37.2 (22.8) |
| Moving, *% (sd)* | 11.7 (5.6) |
|  |  |
| Changes to work after COVID-19 restrictions |  |
| Work hours, *mean (sd)* | -1.44 (5.5) |
| Work location |  |
| Work from home, *n (%)* | 6 (66.7) |
| Mostly work from home, *n (%)* | 1 (11.1) |
| About equal work from home and workplace, *n (%)* | 1 (11.1) |
| Mostly work at workplace, *n (%)* | 1 (11.1) |
| At workplace, *n (%)* | 0 |
| Self-reported change in behaviours in workday |  |
| Sitting, *% (sd)* | -0.56 (29.4) |
| Standing, *% (sd)* | -0.44 (26.8) |
| Moving, *% (sd)* | 1.00 (7.1) |
| Workload compared to before restrictions |  |
| Slightly more work to do | 1 (11.1) |
| Same amount of work to do | 8 (88.9) |
| Care load compared to before restrictions |  |
| Slightly more caring responsibilities | 2 (22.2) |
| Same amount of caring responsibilities | 4 (44.4) |
| Does not have caring responsibilities (N/A) | 3 (33.3) |
|  |  |
| Table displays mean (standard deviation), or sample number, n (%) | |

Multimedia Appendix 2: Table 1 – Self-reported work factors prior to and after COVID-19 restrictions

### Multimedia Appendix 2: Table 2 – Self-reported changes to physical activity and exercise following COVID-19 restrictions

| Physical activity and exercise after COVID-19 restrictions | n=9 |
| --- | --- |
| Walking indoors, *n (%)*  Doing more | 3 (33.3) |
| Doing less or ceased | 4 (44.4) |
| Running indoors, *n (%)*  Doing more | 0 |
| Doing less or ceased | 3 (33.3) |
| Walking outdoors, *n (%)*  Doing more | 4 (44.4) |
| Doing less or ceased | 5 (55.6) |
| Bushwalking, *n (%)*  Doing more | 1 (11.1) |
| Doing less or ceased | 3 (33.3) |
| Running outdoors, *n (%)*  Doing more | 1 (11.1) |
| Doing less or ceased | 3 (33.3) |
| Swimming, *n (%)*  Doing more | 0 |
| Doing less or ceased | 4 (44.4) |
| Cycling indoors, *n (%)*  Doing more | 0 |
| Doing less or ceased | 4 (44.4) |
| Cycling outdoors, *n (%)* Doing more | 1 (11.1) |
| Doing less or ceased | 4 (44.4) |
| Aerobics, *n (%)*  Doing more | 0 |
| Doing less or ceased | 4 (44.4) |
| Yoga / Pilates, *n (%)*  Doing more | 0 |
| Doing less or ceased | 4 (44.4) |
| Resistance exercise, *n (%)*  Doing more | 1 (11.1) |
| Doing less or ceased | 3 (33.3) |
| Sport, *n (%)*  Doing more | 0 |
| Doing less or ceased | 4 (44.4) |
| Table displays sample number, n (%). Only changes as a result of pandemic restrictions were recorded with this questionnaire. | |

| 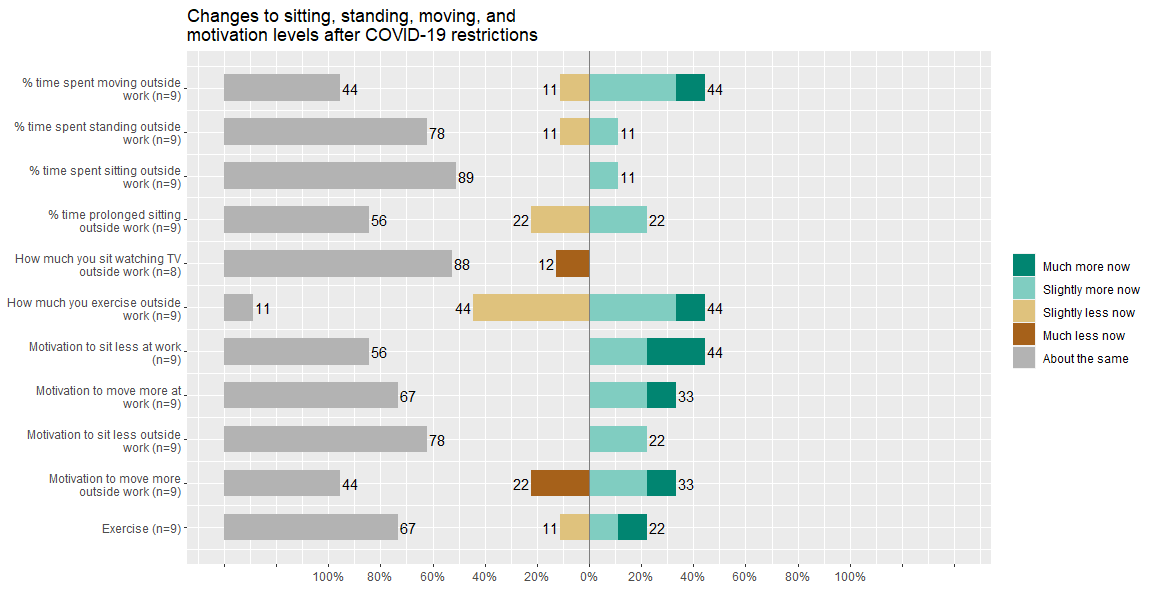 Motivation to |
| --- |
| Figure depicts self-report scale, displays percentage of answers alongside each response in scale. Changes following the COVID-19 pandemic restrictions were surveyed. |

### Multimedia Appendix 2: Figure 1 – Self-reported changes following COVID-19 restrictions on sedentary behaviour and motivation

### Multimedia Appendix 2: Figure 2 – Self-reported changes following COVID-19 restrictions on work environment and access

| 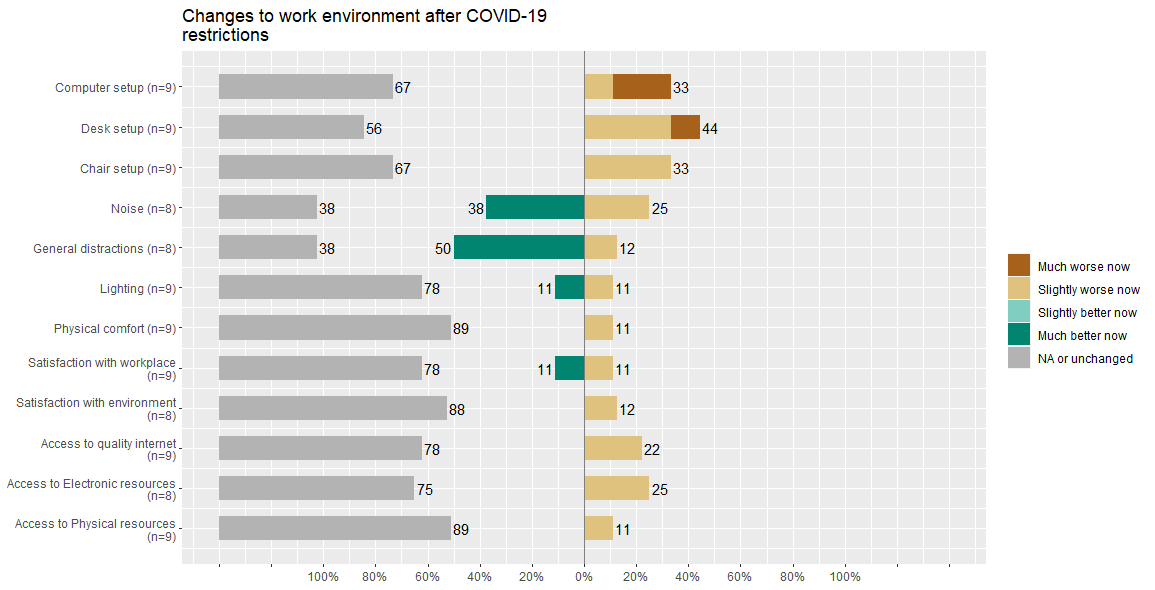 |
| --- |
| Figure depicts self-report scale and displays percentage of answers alongside each response in scale. Changes following the COVID-19 pandemic restrictions were surveyed. |

### Multimedia Appendix 2: Figure 3 – Self-reported changes following COVID-19 restrictions on joint and muscle discomfort

| 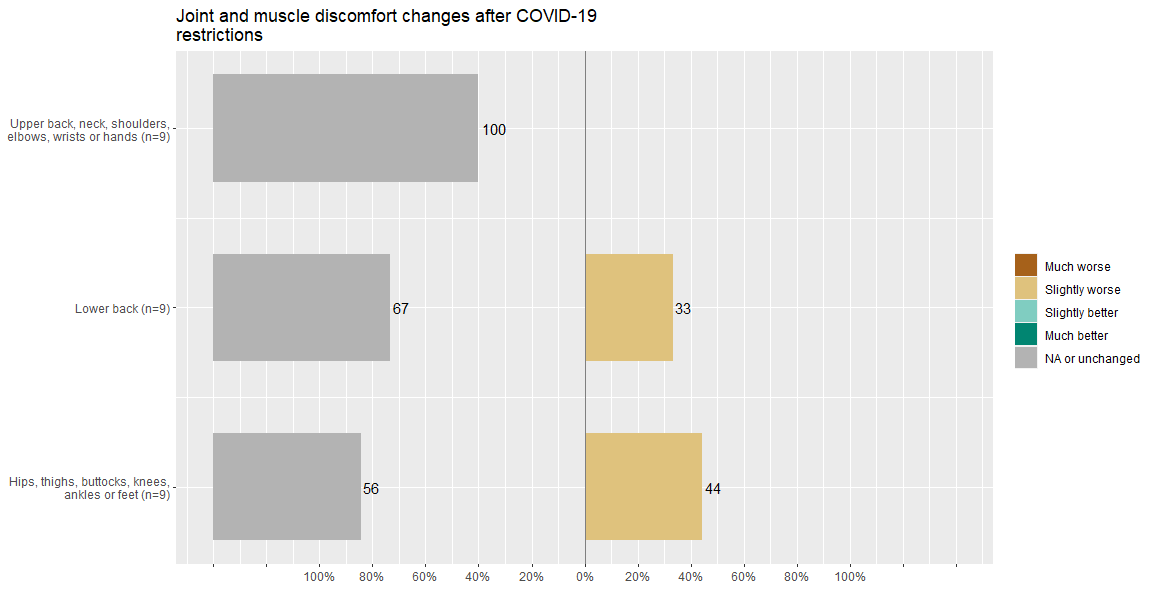 |
| --- |
| Figure depicts self-report scale and displays percentage of answers alongside each response in scale. Changes following the COVID-19 pandemic restrictions were surveyed. |
