# Supplementary material for: The Associations of COVID-19 Lockdown Restrictions With Longer-Term Activity Levels of Working Adults With Type 2 Diabetes: Cohort Study
Source: JMIR Diabetes. 2022 May 18;7(2):e36181. doi: 10.2196/36181 (PMC9119394; doi:10.2196/36181)
Supplement: Multimedia Appendix 3 [file diabetes_v7i2e36181_app3.docx]

## Multimedia Appendix

### *Multimedia Appendix 3– Linear regression activity comparisons prior to lockdown restrictions and during for each participant*

| Multimedia Appendix 3.0 –Comparison of mean steps and metabolic equivalents conducted per day during lockdown restrictions with steps and metabolic equivalents conducted prior to lockdown restrictions | | | | | | | | |
| --- | --- | --- | --- | --- | --- | --- | --- | --- |
|  | Mean steps per day (95% CI) | | | | Mean METs per day (95% CI) | | | |
| Participant | Prior | During |  | RR^a^ (95% CI) | Prior | During |  | RR^a^ (95% CI) |
| ID1 | 12557 (12232, 12891) | 12046 (11681, 12425) | **↓** | 0.96 (0.92, 1.00) | 2198 (2184, 2212) | 2070 (2055, 2084) | **↓** | 0.94 (0.93, 0.95) |
| ID2 | 11284 (10633, 11993) | 10351 (9475, 11329) | **↓** | 0.92 (0.82, 1.02) | 1894 (1877, 1910) | 1838 (1814, 1860) | **↓** | 0.97 (0.96, 0.99) |
| ID3 | 5600 (5258, 5955) | 2987 (2246, 3953) | **↓** | 0.53 (0.39, 0.71) | 1770 (1758, 1782) | 1670 (1628, 1716) | **↓** | 0.94 (0.92, 0.97) |
| ID4 | 13455 (12861, 14066) | 13705 (13055, 14436) | ↑ | 1.02 (0.95, 1.09) | 2176 (2152, 2200) | 2108 (2069, 2147) | **↓** | 0.97 (0.95, 0.99) |
| ID5 | 5918 (5572, 6287) | 3048 (2242, 4157) | **↓** | 0.52 (0.37, 0.71) | 1603 (1589, 1617) | 1536 (1495, 1579) | **↓** | 0.96 (0.93, 0.99) |
| ID6 | 21813 (20418, 23174) | 12361 (11165, 13777) | **↓** | 0.57 (0.51, 0.64) | 2493 (2461, 2523) | 1953 (1929, 1977) | **↓** | 0.78 (0.77, 0.80) |
| ID7 | 6588 (6174, 7011) | 7177 (6567, 7836) | ↑ | 1.09 (0.98, 1.21) | 1695 (1680, 1711) | 1753 (1736, 1772) | ↑ | 1.03 (1.02, 1.05) |
| ID8 | 7458 (7053, 7906) | 7950 (7505, 8411) | ↑ | 1.07 (0.98, 1.16) | 1685 (1671,1700) | 1607 (1592, 1624) | **↓** | 0.95 (0.94, 0.97) |
| ID9 | 10697 (9988, 11401) | 9739 (8891, 10553) | **↓** | 0.91 (0.81, 1.01) | 1937 (1920, 1956) | 1871 (1852, 1894) | **↓** | 0.97 (0.95, 0.98) |
| ID10 | 9066 (8479, 9712) | 9626 (9041, 10298) | ↑ | 1.06 (0.96, 1.16) | 1760 (1745, 1775) | 1787 (1770, 1804) | ↑ | 1.02 (1.00, 1.03) |
| ID11 | 12420 (10813, 14115) | 10437 (10007, 10865) | **↓** | 0.84 (0.73, 0.97) | 2127 (2086, 2170) | 1931 (1917, 1945) | **↓** | 0.91 (0.89, 0.93) |
| Pooled-estimate (SD) | 10623 (4439) | 9039 (3351) | **↓** | 0.91 (0.89, 0.93) | 1940 (264) | 1857 (173) | **↓** | 0.95 (0.94, 0.95) |
| ^a^RR – relative rates are representative of the antilog of the model regression coefficient associated with lockdown restrictions relative to prior lockdown restrictions. RR values >1.00 indicate an increase in activity, and RR values <1.00 indicate a decrease in activity following the lockdown restrictions.  Pooled-estimate determined with fixed-effect meta-analysis and by combining within-participant estimates  METs – Metabolic equivalents per minute; SD – standard deviation | | | | | | | | |

| Multimedia Appendix 3.1 - Comparison of mean intensity minutes per day during lockdown restrictions to minutes conducted per day prior to lockdown restrictions: lightly active intensity physical activity and sedentary minutes | | | | | | | | |
| --- | --- | --- | --- | --- | --- | --- | --- | --- |
|  | Mean lightly active intensity minutes per day (SD) | | | | Mean sedentary minutes per day (SD) | | | |
| Participant | Prior | During |  | RR^a^ (95% CI) | Prior | During |  | RR^a^ (95% CI) |
| ID1 | 318 (13) | 291 (12) | **↓** | 0.92 (0.78, 1.07) | 1007(40) | 1050 (43) | ↑ | 1.05 (0.93, 1.17) |
| ID2 | 220 (14) | 203 (18) | **↓** | 0.92 (0.69, 1.22) | 1101 (69) | 1102 (96) | ↑ | 0.99 (0.81, 1.22) |
| ID3 | 207 (21) | 122 (26) | **↓** | 0.59 (0.29, 1.20) | 1066 (96) | 870 (198) | **↓** | 0.82 (0.52, 1.30) |
| ID4 | 308 (15) | 379 (29) | ↑ | 1.23 (0.97, 1.57) | 947 (47) | 870 (68) | **↓** | 0.92 (0.77, 1.09) |
| ID5 | 139 (7) | 113 (16) | **↓** | 0.81 (0.55, 1.20) | 1275 (68) | 1824 (252) | ↑ | 1.43 (1.07, 1.90) |
| ID6 | 355 (21) | 306 (18) | **↓** | 0.86 (0.68, 1.10) | 866 (51) | 1014 (64) | ↑ | 1.17 (0.99, 1.38) |
| ID7 | 190 (15) | 262 (24) | ↑ | 1.38 (1.00, 1.90) | 1181 (93) | 1141 (108) | **↓** | 0.97 (0.77, 1.22) |
| ID8 | 220 (14) | 169 (12) | **↓** | 0.77 (0.59, 1.00) | 1142 (71) | 1178 (82) | ↑ | 1.03 (0.86, 1.24) |
| ID9 | 221 (17) | 213 (19) | **↓** | 0.96 (0.70, 1.32) | 1118 (89) | 1115 (99) | **↓** | 1.00 (0.79, 1.25) |
| ID10 | 273 (16) | 288 (19) | ↑ | 1.05 (0.83, 1.34) | 1100 (63) | 1048 (67) | **↓** | 0.95 (0.81, 1.13) |
| ID11 | 301 (48) | 288 (16) | **↓** | 0.96 (0.62, 1.49) | 925 (139) | 1037 (56) | ↑ | 1.12 (0.82, 1.54) |
| Pooled-estimate | 251 (6) | 240 (6) | **↓** | 0.96 (0.92, 0.99) | 1064 (25) | 1115 (36) | ↑ | 1.03 (1.01, 1.06) |
| ^a^RR – relative rates are representative of the antilog of the model regression coefficient associated with lockdown restrictions relative to prior lockdown restrictions. RR values >1.00 indicate an increase in activity, and RR values <1.00 indicate a decrease in activity following the lockdown restrictions.  Pooled-estimate determined with fixed-effect meta-analysis and by combining within-participant estimates  SD – standard deviation | | | | | | | | |

| Multimedia Appendix 3.2 - Comparison of mean intensity minutes during lockdown restrictions to minutes conducted prior to lockdown restrictions: fairly active and very active intensity physical activity | | | | | | | | |
| --- | --- | --- | --- | --- | --- | --- | --- | --- |
|  | Mean fairly active intensity minutes per day (SD) | | | | Mean very active intensity minutes per day (SD) | | | |
| Participant | Prior | During |  | RR^a^ (95% CI) | Prior | During |  | RR^a^ (95% CI) |
| ID1 | 21 (1) | 23 (1) | ↑ | 1.07 (0.91, 1.26) | 26 (1) | 26 (1) | **↓** | 0.98 (0.84, 1.16) |
| ID2 | 18 (1) | 15 (1) | **↓** | 0.85 (0.63, 1.15) | 30 (2) | 34 (3) | ↑ | 1.14 (0.85, 1.54) |
| ID3 | 15 (2) | 17 (10) | ↑ | 1.14 (0.37, 3.48) | 5 (0) | 17 (19) | ↑ | 3.32 (0.71, 15.62) |
| ID4 | 20 (1) | 13 (1) | **↓** | 0.64 (0.50, 0.82) | 55 (3) | 36 (3) | **↓** | 0.66 (0.51, 0.85) |
| ID5 | 8 (1) | 5 (1) | **↓** | 0.63 (0.34, 1.15) | 24 (1) | 13 (3) | **↓** | 0.54 (0.31, 0.96) |
| ID6 | 11 (1) | 6 (1) | **↓** | 0.55 (0.43, 0.71) | 123 (7) | 73 (6) | **↓** | 0.59 (0.46, 0.77) |
| ID7 | 16 (1) | 19 (2) | ↑ | 1.24 (0.86, 1.79) | 7 (1) | 5 (1) | **↓** | 0.75 (0.51, 1.10) |
| ID8 | 10 (1) | 7 (1) | **↓** | 0.67 (0.51, 0.88) | 15 (1) | 20 (1) | ↑ | 1.30 (0.98, 1.72) |
| ID9 | 14 (1) | 15 (2) | ↑ | 1.10 (0.79, 1.53) | 26 (2) | 32 (3) | ↑ | 1.25 (0.89, 1.74) |
| ID10 | 5 (0) | 4 (0) | **↓** | 0.75 (0.58, 0.98) | 23 (2) | 26 (2) | ↑ | 1.12 (0.86, 1.45) |
| ID11 | 35 (6) | 15(1) | **↓** | 0.44 (0.27, 0.71) | 23 (4) | 19 (1) | **↓** | 0.83 (0.51, 1.35) |
| Pooled-estimate | 16 (0) | 13 (1) | **↓** | 0.82 (0.79, 0.85) | 32 (1) | 27 (2) | **↓** | 0.92 (0.89, 0.95) |
| ^a^RR – relative rates are representative of the antilog of the model regression coefficient associated with lockdown restrictions relative to prior lockdown restrictions. RR values >1.00 indicate an increase in activity, and RR values <1.00 indicate a decrease in activity following the lockdown restrictions.  Pooled-estimate determined with fixed-effect meta-analysis and by combining within-participant estimates  SD – standard deviation | | | | | | | | |

| Multimedia Appendix 3.3 - Comparison of usual bout durations during lockdown restrictions to bout durations prior to lockdown restrictions: lightly active intensity physical activity and sedentary bouts | | | | | | | | | | | | | | | | |  |
| --- | --- | --- | --- | --- | --- | --- | --- | --- | --- | --- | --- | --- | --- | --- | --- | --- | --- |
|  | | Lightly active intensity bout minutes (SD) | | | | | | | | Sedentary bout minutes (SD) | | | | | | |  |
| Participant | | Prior | | During | |  | | RR^a^ (95% CI) | | Prior | | During | |  | | RR^a^ (95% CI) |  |
| ID1 | | 5 (0.1) | | 5 (0.2) | | ↑ | | 1.01 (0.93, 1.10) | | 15.2 (0.4) | | 20.1 (0.5) | | ↑ | | 1.32 (1.22, 1.42) |  |
| ID2 | | 4.2 (0.4) | | 5.3 (0.8) | | ↑ | | 1.26 (0.91, 1.72) | | 25.3 (0.6) | | 32.6 (1.0) | | ↑ | | 1.29 (1.19, 1.39) |  |
| ID3 | | 4 (0.1) | | 3.1 (0.3) | | **↓** | | 0.78 (0.64, 0.95) | | 24.3 (0.7) | | 33.8 (4.2) | | ↑ | | 1.38 (1.07, 1.77) |  |
| ID4 | | 5 (0.1) | | 5.7 (0.5) | | ↑ | | 1.13 (0.96, 1.35) | | 15.4 (0.5) | | 17.3 (0.5) | | ↑ | | 1.13 (1.04, 1.23) |  |
| ID5 | | 4 (0.0) | | 3.9 (0.4) | | **↓** | | 0.97 (0.79, 1.18) | | 33 (1.1) | | 34.7 (2.0) | | ↑ | | 1.05 (0.92, 1.20) |  |
| ID6 | | 4 (0.1) | | 4 (0.0) | | - | | 1.00 (0.97, 1.03) | | 14.2 (0.5) | | 19.8 (0.7) | | ↑ | | 1.40 (1.27, 1.54) |  |
| ID7 | | 4.8 (0.4) | | 5.9 (0.2) | | ↑ | | 1.24 (1.02, 1.51) | | 26.1 (0.8) | | 29.1 (1.3) | | ↑ | | 1.11 (1.00, 1.24) |  |
| ID8 | | 4 (0.0) | | 4 (0.0) | | - | | 1.00 (1.00, 1.00) | | 21.9 (0.6) | | 30.1 (0.7) | | ↑ | | 1.38 (1.29, 1.47) |  |
| ID9 | | 4 (0.0) | | 4 (0.0) | | - | | 1.00 (1.00, 1.00) | | 18.5 (0.6) | | 25 (1.1) | | ↑ | | 1.35 (1.21, 1.50) |  |
| ID10 | | 4 (0.1) | | 4 (0.2) | | **↓** | | 0.99 (0.89, 1.10) | | 15.6 (0.6) | | 13.4 (0.5) | | **↓** | | 0.86 (0.77, 0.95) |  |
| ID11 | | 5.3 (0.5) | | 4.7 (0.5) | | **↓** | | 0.88 (0.68, 1.14) | | 12.7 (0.6) | | 14.2 (0.4) | | ↑ | | 1.12 (1.00, 1.25) |  |
| Pooled-estimate | | 4.4 (0.1) | | 4.5 (0.1) | | ↑ | | 1.01 (0.96, 1.07) | | 20.2 (0.2) | | 24.6 (0.5) | | ↑ | | 1.20 (1.16, 1.25) |  |
| ^a^RR – relative rates are representative of the antilog of the model regression coefficient associated with lockdown restrictions relative to prior lockdown restrictions. RR values >1.00 indicate an increase in activity, and RR values <1.00 indicate a decrease in activity following the lockdown restrictions.  Usual bout duration describes the median weighted bout length; participants accumulate half of all their activity time in bouts longer than the estimate  Pooled-estimate determined with fixed-effect meta-analysis and by combining within-participant estimates  SD – standard deviation | | | | | | | | | | | | | | | | |  |
| Multimedia Appendix 3.4 - Comparison of usual bout durations during lockdown restrictions to bout durations conducted prior to lockdown restrictions: fairly active and very active intensity physical activity bouts | | | | | | | | | | | | | | | | | |
|  | Fairly active intensity bout minutes (SD) | | | | | | | | Very active intensity bout minutes (SD) | | | | | | | | |
| Participant | Prior | | During | |  | | RR^a^ (95% CI) | | Prior | | During | |  | | RR^a^ (95% CI) | | |
| ID1 | 3 (0.1) | | 2.2 (0.4) | | **↓** | | 0.73 (0.52, 1.03) | | 6 (0.2) | | 6.2 (0.4) | | ↑ | | 1.03 (0.90, 1.17) | | |
| ID2 | 3 (0.1) | | 3 (0.0) | | - | | 1 (0.95, 1.06) | | 10.3 (0.8) | | 23.8 (1.7) | | ↑ | | 2.32 (1.89, 2.86) | | |
| ID3 | 3 (0.2) | | 3 (0.6) | | **↓** | | 0.96 (0.64, 1.42) | | 9 (2.4) | | 7.6 (3.5) | | **↓** | | 0.76 (0.22, 2.58) | | |
| ID4 | 2 (0.0) | | 2 (0.0) | | - | | 1 (1.00, 1.00) | | 8.2 (0.4) | | 8 (0.2) | | **↓** | | 0.99 (0.89, 1.09) | | |
| ID5 | 2 (0.1) | | 2.1 (1.1) | | **↓** | | 0.95 (0.35, 2.57) | | 9.5 (0.6) | | 20.8 (11.6) | | ↑ | | 1.84 (0.52, 6.51) | | |
| ID6 | 2 (0.1) | | 2.2 (0.4) | | ↑ | | 1.06 (0.79, 1.44) | | 78.4 (4.9) | | 97.4 (9.5) | | ↑ | | 1.24 (0.99, 1.56) | | |
| ID7 | 3.1 (0.3) | | 3.8 (0.4) | | ↑ | | 1.2 (0.90, 1.61) | | 3.4 (0.5) | | 3.6 (0.5) | | ↑ | | 1.05 (0.7, 1.58) | | |
| ID8 | 2.9 (0.2) | | 2.1 (0.3) | | **↓** | | 0.71 (0.53, 0.94) | | 10.6 (1.9) | | 13.4 (1.0) | | ↑ | | 1.28 (0.89, 1.85) | | |
| ID9 | 3 (0.2) | | 3 (0.0) | | ↑ | | 1.01 (0.88, 1.18) | | 17.8 (2) | | 17.5 (1.5) | | **↓** | | 0.99 (0.74, 1.31) | | |
| ID10 | 1.9 (0.2) | | 2.1 (1.9) | | **↓** | | 0.99 (0.44, 2.19) | | 17.9 (2.1) | | 16.5 (1.4) | | **↓** | | 0.93 (0.7, 1.22) | | |
| ID11 | 4 (0.2) | | 3 (0.1) | | **↓** | | 0.75 (0.67, 0.83) | | 4.9 (1.1) | | 10.9 (1.0) | | ↑ | | 2.29 (1.44, 3.63) | | |
| Pooled-estimate | 2.7 (0.1) | | 2.6 (0.2) | | **↓** | | 0.93 (0.81, 1.06) | | 16 (0.6) | | 20.5 (1.4) | | ↑ | | 1.25 (1.04, 1.49) | | |
| ^a^RR – relative rates are representative of the antilog of the model regression coefficient associated with lockdown restrictions relative to prior lockdown restrictions. RR values >1.00 indicate an increase in activity, and RR values <1.00 indicate a decrease in activity following the lockdown restrictions.  Usual bout duration describes the median weighted bout length; participants accumulate half of all their activity time in bouts longer than the estimate  Pooled-estimate determined with fixed-effect meta-analysis and by combining within-participant estimates  SD – standard deviation | | | | | | | | | | | | | | | | | |
